# Longitudinal changes in each retinal layer thickness in patients with moderate or more severe diabetic retinopathy taking calcium dobesilate

**DOI:** 10.1371/journal.pone.0325655

**Published:** 2025-06-04

**Authors:** Jung-Tae Kim, Min-Woo Lee

**Affiliations:** Department of Ophthalmology, Konyang University College of Medicine, Daejeon, Republic of Korea; Akita University: Akita Daigaku, JAPAN

## Abstract

**Purpose:**

To longitudinally analyze the impact of calcium dobesilate (CaD) treatment on retinal layer thicknesses in patients with moderate or more severe diabetic retinopathy (DR).

**Methods:**

In this retrospective, longitudinal study, patients with DR exhibiting moderate non-proliferative diabetic retinopathy (NPDR) or more advanced stages were enrolled and divided into two groups: those prescribed CaD for more than 6 months (Group 1) and those not prescribed CaD (Group 2). After the baseline visits, three additional examinations were conducted at 1-year intervals.

**Results:**

In total, 128 eyes were included: 38 in Group 1 and 90 in Group 2. Over time, group 2 exhibited significant decreases in ganglion cell complex (GCC) and inner nuclear layer (INL) thicknesses (P < 0.001 and P = 0.002, respectively), while Group 1 did not. Both groups experienced significant reductions in outer nuclear layer (ONL) thickness (P = 0.030 for Group 1 and P < 0.001 for Group 2). Group 2 exhibited a significantly greater decrease in GCC thickness compared with Group 1(P = 0.026).

**Conclusions:**

In patients with moderate or more severe DR, the group taking CaD did not show significant reductions in GCC and INL thicknesses over time compared with the group not taking CaD. These findings suggest that CaD could have a protective effect on the inner retina, even in those with more advanced stages than mild DR.

## Introduction

Diabetic retinopathy (DR) is a major cause of visual impairment in working-age adults worldwide [1,2]. Current treatments include intravitreal steroid or anti-vascular endothelial growth factor (VEGF) injections and pan-retinal photocoagulation (PRP). However, these treatments are invasive, expensive, and applicable only at advanced DR when significant visual impairment has already occurred. When the early stages of DR are the therapeutic target, it would be inconceivable to recommend an aggressive treatment, and pharmacological treatment to slow or stop its progression would be desirable.

Calcium dobesilate (CaD) has been widely prescribed for many years to prevent DR progression. It is recognized for its angio-protective properties and its positive effects on the inner blood-retinal barrier (BRB) [3]. CaD is known to reduce microvascular permeability through its antioxidant properties and enhances endothelium-dependent relaxation by promoting nitric oxide synthesis. Experimental and pharmacological evidence suggests its potential to stabilize the BRB in DR by reducing oxidative stress and vascular permeability [4]. Similarly, in clinical trials, CaD slowed early DR progression by exerting anti-inflammatory, antioxidant, and antiangiogenic effects, and by enhancing endothelial-dependent vasodilation [5,6]. Other studies also show that CaD reduced retinal microaneurysms, retinal hemorrhages, exudates, and blood viscosity in DR [6–8]. However, most previous studies focused on patients with early DR; few works have explored the effectiveness of CaD in those with moderate or more severe DR.

Diabetic retinal neurodegeneration (DRN) can occur persistently across a wide range of stages, from diabetic patients where DR is not yet observed to those with advanced DR. DRN primarily affects the inner retinal layers, driven by chronic hyperglycemia, oxidative stress, and accumulation of advanced glycation end-products; these changes increase glutamate levels and trigger the loss of neuroprotective factors [9–13]. Previous studies showed that CaD exerted both vasculotropic and neurotropic actions, including antioxidative, anti-inflammatory effects, and prevention of glial activation and apoptosis. In addition, CaD prevents glutamate accumulation, and the inhibitory effect of CaD on ET-1 could also contribute to neuroprotection [14–17]. Thus, we hypothesized that CaD would be beneficial in DRN, although any effect of CaD on inner retinal layer thickness in patients with DR has not been longitudinally examined.

This study aims to longitudinally analyze the effects of CaD on the thicknesses of various retinal layers in patients with moderate or more severe DR and confirm the effect of CaD in relation to DRN progression.

## Methods

### Patients

This retrospective, longitudinal, observational study received approval from the Institutional Review Board/Ethics Committee of Konyang University Hospital, Daejeon, Republic of Korea (No. 2024-09-015) and was conducted in accordance with the principles of the Declaration of Helsinki. The data for research purposes wre assessed on October 18, 2024. We reviewed the medical records of patients diagnosed with DR who had undergone checkups from March 2017 to December 2023 in our retinal clinic. Patients with DR who have moderate non-proliferative diabetic retinopathy(NPDR) or more advanced stages were enrolled. The requirement for informed consent was waived by the Institutional Review Board/Ethics Committee of Konyang University Hospital due to the retrospective nature of the study. We retrieved best-corrected visual acuity (BCVA), intraocular pressure, spherical equivalent, axial length, and spectral-domain optical coherence tomography (SD-OCT) data. Patients were divided into two groups: those receiving 1 g CaD daily for more than 6 months (Group 1) and those not prescribed CaD (Group 2). After the baseline visit, three additional examinations were conducted at 1-year intervals.

The exclusion criteria included a history of any retinal disease other than DR, mild NPDR, severe cataracts significantly affecting vision, glaucoma, any intraocular surgery with the exception of cataract extraction, an intraocular pressure ≥ 21 mmHg, an axial length ≥ 26 mm, and a BCVA < 20/40. We also excluded patients who had undergone anti-VEGF injection treatment either before baseline or during the follow-up period, along with those who had undergone PRP within the past six months, as these factors may affect retinal layer thickness. Additionally, patients who had received any treatment for DR other than PRP or CaD prescription prior to baseline, as well as those who underwent any ophthalmic treatment for DR during the follow-up period, were also excluded. During the follow-up period, patients who exhibited a central macular thickness ≥ 300 μm, cystic macular changes, or DR deterioration requiring PRP, as well as those who underwent cataract surgery, were excluded. If both eyes met the inclusion criteria, one eye was randomly selected to avoid statistical bias.

### Retinal layer thickness analysis

**Fig 1:**
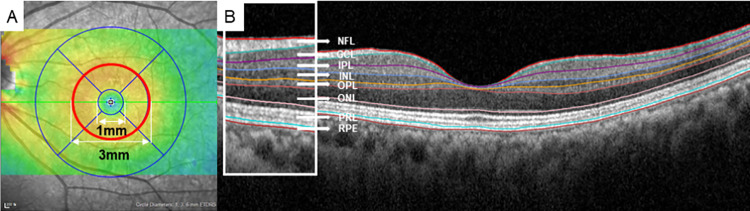
Macular regions analyzed by spectral-domain optical coherence tomography scan in the retinal thickness map analysis. A. The parafoveal thickness, defined as the area within a 1 to 3 mm radius from the subfoveal region (red circle), was analyzed. B. Representative B-scan image. NFL, nerve fiber layer; GCL, ganglion cell layer; IPL, inner plexiform layer; INL, inner nuclear layer; OPL, outer plexiform layer; ONL, outer nuclear layer; PRL, photoreceptor layer; RPE, retinal pigment epithelium.

SD-OCT measurements were performed by a skilled examiner using the Spectralis OCT2 platform (Heidelberg Engineering, Heidelberg, Germany), with built-in segmentation software in Heidelberg Eye Explorer version 6.9a (Heidelberg Engineering). Each volume scan featured 25 horizontal line scans (512 a-scans per b-scan, 245 μm interscan distance) with automatic real-time averaging six images. Numeric averages of measurements for the nine Early Treatment Diabetic Retinopathy Study (ETDRS) subfields were established through retinal thickness map analyses to assess retinal layer thickness. We conducted an analysis of the parafoveal area—defined as the intermediate ring of ETDRS subfields (1–3 mm from the subfoveal region)—to comprehensively characterize changes over time, noting its relatively thicker inner retinal layer. Built-in Heiderberg Eye Explorer ver. 6.9a software (Heidelberg Engineering) was used for automated retinal layer segmentation. Measurements were taken for the thicknesses of the ganglion cell complex (GCC, comprising the retinal nerve fiber layer, ganglion cell layer, and inner plexiform layer), as well as the inner nuclear layer (INL), outer plexiform layer (OPL), outer nuclear layer (ONL), photoreceptor layer (PRL), and retinal pigment epithelium (RPE), with these measurements having been previously shown to be highly reliable (Fig 1) [18]. All OCT images were reviewed by two independent investigators (J.T.K. and M.W.L.), and manual adjustments were made when clear segmentation errors were identified. Images with quality scores < 15 were excluded, as were those exhibiting decentration, misalignment, or severe segmentation errors.

### Statistical analysis

Baseline demographic characteristics and ocular parameters were compared with the independent t-test. The Chi-squared test was utilized to compare categorical variables. An ANCOVA was performed to control for HbA1c level (which was significantly different between the two groups), duration of type 2 diabetes, and hypertension as covariates when comparing each retinal layer at baseline. To identify significant within-group changes over time and the rate of thickness reduction in each retinal layer, linear mixed models were applied. Each retinal layer thickness was fitted with linear mixed models, with the HbA1c level as a fixed effect to control for its influence. In Group 1, the duration of CaD intake was also included as a fixed effect. A random intercept was included at the eye level. Generalized linear mixed models were used to identify factors associated with changes in BCVA over time; these values were multiplied by 100 prior to analysis. We also analyzed the factors influencing the retinal layers that showed significant thickness changes in each group. Statistical significance was defined as a P-value of less than 0.050. All statistical calculations were performed with SPSS Statistics software (version 18.0; IBM Corp., Armonk, NY, USA).

## Results

### Baseline demographics

**Fig 2:**
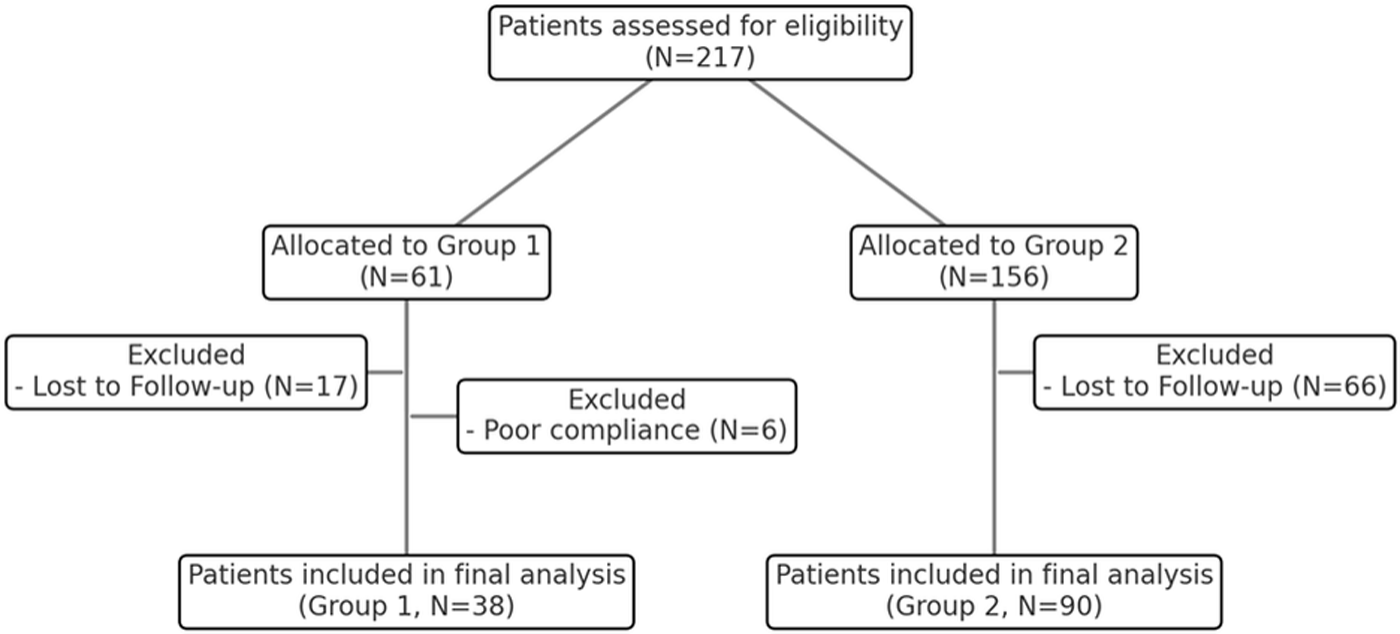
Flow diagram showing number of subjects included in the study population. From the screening data, which excluded patients with exclusion critieria (e.g., ophthalmic diseases or a history of prior treatment for DR such as anti-VEGF injection, among others), we further excluded those with missing data or poor compliance with calcium dobesilate intake as recorded in the charts.

In total, 128 eyes were included: 38 in Group 1, 90 in Group 2 (Table 1) (Fig 2).

**Table 1:** Baseline demographic characteristics.

	Group 1 (n = 38)	Group 2 (n = 90)	P value
Age (years, mean ± SD)	59.2 ± 9.4	57.5 ± 7.2	0.269
Sex (male, %)	19 (50.0)	47 (52.2)	0.818
T2DM duration (years, mean ± SD)	14.0 ± 6.7	14.6 ± 7.4	0.681
HbA1c (%, mean ± SD)	7.5 ± 1.4	8.4 ± 1.7	**0.011**
Hypertension (n, %)	12 (31.6)	34 (37.8)	0.174
SE (diopters, mean ± SD)	−0.96 ± 1.53	−0.50 ± 1.39	0.066
IOP (mmHg, mean ± SD)	14.5 ± 3.3	15.3 ± 3.0	0.219
AXL (mm, mean ± SD)	23.4 ± 0.7	23.2 ± 0.4	0.134
BCVA (logMAR, mean ± SD)	0.04 ± 0.08	0.05 ± 0.08	0.683
CMT (μm, mean ± SD)	269.1 ± 31.3	267.9 ± 25.0	0.833

T2DM, type 2 diabetes mellitus; SE, spherical equivalent; IOP, intraocular pressure; AXL, axial length; BCVA, best-corrected visual acuity; CMT, central macular thickness.

Values in boldface (P < 0.050) are statistically significant.

The mean ages were 59.2 ± 9.4 years in Group 1 and 57.5 ± 7.2 years in Group 2 (P = 0.269). The HbA1c levels were 7.5 ± 1.4, 7.4 ± 1.4, 7.3 ± 1.4, and 7.5 ± 1.6% in Group 1, and 8.4 ± 1.7, 7.9 ± 1.6, 7.8 ± 1.5, and 7.8 ± 1.6% in Group 2, which was significantly different between the groups at baseline (P = 0.011). The number of patients with NPDR of at least a moderate stage but not requiring PRP was 21 (moderate NPDR, 9; severe NPDR, 12) in Group 1 and 63 (moderate NPDR, 25; severe NPDR, 38) in Group 2 (P = 0.604). Meanwhile, the number of patients with very severe NPDR or PDR who had previously undergone PRP was 17 in Group 1 and 27 in Group 2 (P = 0.129). Other factors, including sex, duration of type 2 diabetes mellitus, hypertension status, spherical equivalent, intraocular pressure, axial length, BCVA, and central macular thickness (CMT) were not significantly different between the groups. The average duration of CaD intake at baseline in Group 1 was 11.6 ± 5.4 months (Max: 26 months, Min: 6 months). Over time, both groups showed a tendency for BCVA deterioration. However, in Group 1, the change was not statistically significant (P = 0.197), whereas in Group 2, it was statistically significant (P < 0.001). Despite this, the difference in changes between the two groups was not statistically significant (P = 0.783).

### Parafoveal thickness of each retinal layer at each visit

At baseline, there were no significant differences between the groups in any of GCC, INL, OPL, ONL, PRL, or RPE thickness (Table 2).

**Table 2:** Parafoveal thickness in each retinal layer at each visit.

	Group 1	Group 2	P value^*^
GCC			
Baseline	114.3 ± 15.1	113.7 ± 16.0	0.627
First year	113.4 ± 14.4	111.9 ± 15.5	
Second year	113.3 ± 15.7	110.7 ± 15.9	
Third year	112.7 ± 17.4	109.9 ± 15.8	
P-value^†^	0.409	**< 0.001**	
INL			
Baseline	41.3 ± 3.9	42.1 ± 3.6	0.377
First year	40.9 ± 3.9	41.7 ± 3.4	
Second year	40.9 ± 4.1	41.6 ± 3.6	
Third year	40.8 ± 4.1	41.3 ± 3.7	
P-value^†^	0.188	**0.002**	
OPL			
Baseline	35.8 ± 6.7	35.1 ± 5.7	0.773
First year	36.1 ± 6.4	35.5 ± 5.8	
Second year	35.6 ± 6.7	34.9 ± 5.9	
Third year	36.6 ± 7.5	35.5 ± 5.8	
P-value^†^	0.838	0.517	
ONL			
Baseline	70.9 ± 11.4	70.4 ± 10.0	0.310
First year	68.7 ± 10.8	68.4 ± 9.6	
Second year	67.4 ± 11.1	66.8 ± 9.9	
Third year	66.8 ± 12.6	66.6 ± 9.9	
P-value^†^	**0.030**	**< 0.001**	
PRL			
Baseline	65.5 ± 2.1	65.8 ± 2.4	0.936
First year	66.0 ± 1.9	66.0 ± 2.1	
Second year	66.0 ± 2.1	66.0 ± 2.0	
Third year	65.3 ± 2.2	66.2 ± 2.3	
P-value^†^	0.058	0.074	
RPE			
Baseline	15.0 ± 1.1	14.8 ± 1.3	0.432
First year	15.2 ± 1.4	14.8 ± 1.5	
Second year	15.1 ± 1.2	14.8 ± 1.4	
Third year	15.0 ± 1.2	14.8 ± 1.4	
P-value^†^	0.863	0.411	

GCC, ganglion cell complex; INL, inner nuclear layer; OPL, outer plexiform layer; ONL, outer nuclear layer; PRL, photoreceptor layer; RPE, retinal pigment epithelium.

Values in boldface (P < 0.050) are statistically significant.

* ANCOVA for baseline values.

† Linear mixed model.

**Fig 3:**
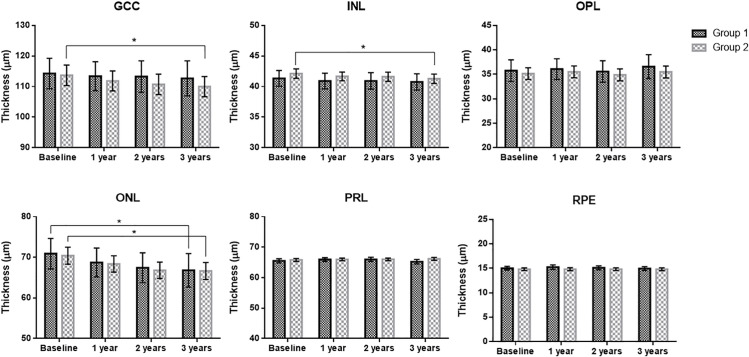
Bar graphs showing the means and 95% CI of each retinal layer thickness at each visit. Ganglion cell complex (GCC) and inner nuclear layer (INL) thicknesses in Group 2, and the outer nuclear layer (ONL) thickness in both groups showed significant changes over time. OPL, outer plexiform layer; PRL, photoreceptor layer; RPE, retinal pigment epithelium.

Over time, Group 2 showed significant decreases in GCC and INL thickness (P < 0.001 and P = 0.002, respectively), while Group 1 did not. Both groups displayed significant reductions in ONL thickness (P = 0.030 for group 1 and P < 0.001 for group 2). There were no significant changes over time in the OPL, PRL, or RPE thicknesses in either group (Fig 3).

### Rate of change in each retinal layer thickness and associations with BCVA changes

Group 2 exhibited a significantly greater decrease in GCC thickness compared with Group 1 (P = 0.026) (Table 3).

**Table 3:** Rates of change in each retinal layer thickness, calculated using linear mixed models.

	Group 1	Group 2	P value^*^
GCC	−0.36 (−1.24 to 0.51)	−1.33 (−1.61 to −1.04)	**0.008**
INL	−0.19 (−0.47 to 0.10)	−0.31 (−0.51 to −0.11)	0.574
OPL	0.06 (−0.50 to 0.62)	0.10 (−0.21 to 0.42)	0.790
ONL	−0.99 (−1.89 to −0.10)	−1.02 (−1.52 to −0.52)	0.959
PRL	−0.24 (−0.40 to 0.03)	0.12 (−0.01 to 0.25)	0.061
RPE	0.01 (−0.11 to 0.13)	0.03 (−0.04 to 0.11)	0.619

GCC, ganglion cell complex; INL, inner nuclear layer; OPL, outer plexiform layer; ONL, outer nuclear layer; PRL, photoreceptor layer; RPE, retinal pigment epithelium.

Values in boldface (P < 0.050) are statistically significant.

* Interaction between group and duration in linear mixed models.

There were no significant differences between the groups in the rates of change of other layers. We investigated the factors influencing the thickness changes of GCC, INL, and ONL, which showed significant changes in Group 2. As a result, among various factors such as age, sex, and HTN status, only DM duration had a significant impact on the thickness changes of GCC (estimate = −0.60, P = 0.018) and ONL (estimate = −0.44, P = 0.004) in Group 2. In Group 1, no factors were found to have a significant influence on the thickness changes of GCC, ONL, or INL. An additional subgroup analysis based on PRP status was conducted for the GCC, INL, and ONL. In patients with PRP, Group 2 showed a significantly faster thinning of the GCC compared to Group 1 (P = 0.036), while changes in the INL (P = 0.532) and ONL (P = 0.619) did not differ significantly between the two groups. In patients without PRP, GCC thinning was also significantly faster in Group 2 (P = 0.044), similar to those with PRP, while INL (P = 0.925) and ONL (P = 0.491) showed no significant differences between the two groups.

In univariate analysis, the ONL (estimate = −0.17, P = 0.001) and PRL (estimate = −0.41, P = − 0.047) thicknesses were significantly associated with changes in BCVA (Table 4). In multivariate analysis, the ONL thickness (estimate = −0.18, P < 0.001) remained statistically significant.

**Table 4:** Linear mixed-effect model determination of factors associated with changes in best-corrected visual acuity.

	Univariate		Multivariate	
	Estimate (95% CI)	P value	Estimate (95% CI)	P value
Age	0.19 (−0.01 to 0.32)	0.058		
Sex	−2.27 (−4.82 to 0.28)	0.080		
T2DM duration	0.10 (−0.09 to 0.29)	0.286		
HTN	−0.43 (−3.24 to 2.38)	0.763		
SE	0.49 (−0.56 to 1.54)	0.358		
IOP	0.04 (−0.41 to 0.48)	0.875		
AXL	0.23 (−4.65 to 5.12)	0.923		
PRP status	0.83 (−1.85 to 3.50)	0.542		
CMT	−0.01 (−0.01 to 0.01)	0.742		
GCC	0.02 (−0.06 to 0.01)	0.640		
INL	0.11 (−0.14 to 0.37)	0.388		
OPL	0.12 (−0.03 to 0.28)	0.110		
ONL	−0.17 (−0.27 to −0.07)	**0.001**	−0.18 (−0.28 to −0.08)	**< 0.001**
PRL	−0.41 (−0.81 to −0.01)	**0.047**	−0.43 (−0.85 to 0.01)	0.090
RPE	−0.52 (−1.16 to 0.11)	0.103		

T2DM, type 2 diabetes mellitus; HTN, hypertension; SE, spherical equivalent; IOP, intraocular pressure; AXL, axial length; PRP, pan-retinal photocoagulation; CMT, central macular thickness; GCC, ganglion cell complex; INL, inner nuclear layer; OPL, outer plexiform layer; ONL, outer nuclear layer; PRL, photoreceptor layer; RPE, retinal pigment epithelium.

Values in boldface (P < 0.050) are statistically significant

Subgroup analysis based on PRP status.

## Discussion

While numerous investigations have examined the impact of CaD on the retina in patients with DR, they have not assessed the effects on DRN. Furthermore, the potential role of CaD in advanced stages of DR has not been adequately addressed in the literature. The present study bridged this gap by conducting a longitudinal analysis of retinal layer thickness changes in patients with moderate or more severe DR who were or were not prescribed CaD. Solà-Adell et al. [16] found that oral CaD (200 mg/kg per day for 14 days) inhibited both glial activation and apoptosis in db/db mice, in comparison with diabetic mice treated with a vehicle. CaD also prevents glutamate accumulation by inhibiting diabetes-induced downregulation of the glutamate/l-aspartate transporter (GLAST). Additionally, Kobayashi et al. [17] observed that ET-1 expression enhanced glutamate-induced neurotoxicity in retinal neural cells; inhibition of ET-1 by CaD might thus be neuroprotective. These multifaceted pharmacological effects of CaD suggested that it could alleviate DRN pathophysiology. We found substantial reductions in GCC and INL thicknesses over time in the group that did not receive CaD, but not in the CaD-treated group, suggesting that CaD supplementation may confer a protective effect on the inner retinal layers in patients with advanced DR.

Several studies have reported that DRN reduces inner retinal layer thickness in DR patients. Sohn et al. [19] reported progressive loss of the inner retina, including the RNFL and ganglion cell-inner plexiform layer (GC-IPL), in patients with diabetes mellitus (DM) but minimal to no DR. Similarly, Van Dijk et al. [9] found a significant inverse correlation between the duration of DM and ganglion cell layer (GCL) thickness, suggesting that neurodegeneration was primarily driven by a prolonged disturbance in glucose metabolism, potentially independent of vasculopathy. Similar to the findings of previous studies, in Group 2, DM duration influenced the thickness changes of GCC. However, in Group 1, as the thickness changes of GCC over time became insignificant, the influence of DM duration also diminished, which can be attributed to the effect of CaD.

The significantly reduced rate of GCC thinning within the CaD-treated group in the present study supports the hypothesis that CaD mitigates DRN by preserving inner retinal thickness. Although previous research has highlighted the effectiveness of CaD in early-stage DR patients, it remains unclear whether CaD can effectively treat those with advanced DR [5 6]. In this respect, the significance of this study is that it provides evidence suggesting a potential attenuating effect of CaD on DRN in patients with moderate or more severe DR. However, a limitation is that all patients with severe DR, including very severe NPDR and PDR, had undergone PRP, while those who had not received PRP were at less advanced DR stages. As a result, the direct impact of PRP could not be independently analyzed. Nevertheless, the subgroup analysis in our study demonstrated that CaD exerted a protective effect against DRN regardless of PRP status. While these structural findings are promising, additional studies incorporating functional assessments are warranted to fully elucidate the clinical relevance of these observations.

This study further demonstrated that changes in INL thickness over time were predominantly observed in the group not receiving CaD (Group 2). This could be attributed to the presence of a deep vascular complex within the INL, which may have been better preserved in Group 1 due to the angio-protective effects of CaD. Consequently, significant thinning of the INL was only apparent in Group 2. To validate this hypothesis, it is necessary to demonstrate that various parameters of the deep vascular complex measured via OCT angiography are correlated with INL thickness, along with their improvement through CaD administration. Additionally, it is crucial to note that the difference in thickness change between the two groups did not reach statistical significance, possibly due to the limited sample size in Group 1 or the inherently slow rate of reduction, resulting in a smaller difference. Therefore, further research involving larger sample sizes and more comprehensive analysis using OCT angiography is necessary to validate these findings and elucidate the underlying mechanisms. Incorporating various visual function tests into the analysis could also help determine how this protective effect is clinically related.

In this study, it was confirmed that the thickness of the ONL significantly decreased in both groups. From this, it can be inferred that the administration of CaD did not influence the changes in ONL thickness. As the angio-protective effects of CaD are considered more dominant in the retina than its neuroprotective actions, the inner retinal layers, which are rich in microvasculature, may benefit more visibly. In contrast, the ONL, mainly composed of photoreceptor cell bodies, may show less marked effects. Meanwhile, multivariate analysis revealed a negative correlation between ONL thickness and the BCVA. Many previous studies have reported a significant association between visual acuity and ONL thickness in various retinal diseases, including central retinal vein occlusions, wet age-related macular degeneration, and central serous chorioretinopathy [20–23]. Therefore, the evaluation of ONL thickness is regarded as an important parameter in the assessment of various retinal diseases. As CaD administration did not affect ONL thickness, changes in visual acuity may not have differed between the two groups. Further large-scale studies are needed to investigate the relationship between ONL thickness and visual function, as well as to explore potential methods for protecting the ONL.

This study had several limitations. First, the retrospective design and the difference in sample size between the two groups may have introduced some bias, requiring careful interpretation before drawing generalizations. Due to the inherent limitations of this study design, we were unable to account for various systemic factors that could have influenced the outcomes aside from CaD, such as BMI, cholesterol levels, or changes in internal medicine medications. Additionally, although HbA1c levels were statistically adjusted for, the baseline difference in HbA1c between the two groups may still have influenced the results to some extent. Future large-scale prospective studies are needed, and including a broader range of patients to compare the severity of DR progression and the occurrence of other complications could also be beneficial. Second, the study was conducted on patients with moderate or more severe DR, and further research is needed to include a wider range of DR severities and analyze the effect of CaD at each stage, including diabetic patients without DR. Third, the absence of diverse visual function tests, including visual field tests, electrophysiological tests, color vision evaluations, and contrast sensitivity analyses, precluded any analysis of the relationship between changes in retinal layer thickness and visual function. The strength of this study lies in its focus on the effects of CaD on moderate or more severe DR patients with longitudinal analysis, an area not previously reported. Another important aspect is that this study provides insight into the potential usefulness of CaD in protecting against the reduction of inner retinal thickness, suggesting that CaD may be effective in preventing DRN.

In patients with moderate or more severe DR, the group prescribed CaD did not exhibit reductions in GCC or INL thickness over time, whereas the group not receiving CaD did show these reductions. The rate of GCC thickness reduction was greater in the group not on CaD. Consequently, CaD intake was advantageous for the protection of the inner retina in DR patients, even if DR was more advanced than the mild stage. To more clearly elucidate the effects of CaD on retinal structural changes and various visual functions, future large-scale prospective studies are warranted.

## Supporting information

S1 Data This file contains the complete dataset analyzed in the current study, including all variables used for statistical modeling. (XLSX)
